# Different subpopulations of regulatory T cells in human autoimmune disease, transplantation, and tumor immunity

**DOI:** 10.1002/mco2.137

**Published:** 2022-04-21

**Authors:** Zhongyi Jiang, Haitao Zhu, Pusen Wang, Weitao Que, Lin Zhong, Xiao‐Kang Li, Futian Du

**Affiliations:** ^1^ Department of General Surgery Shanghai General Hospital Shanghai Jiao Tong University School of Medicine Shanghai P. R. China; ^2^ Department of Hepatobiliary Surgery The Affiliated Hospital of Guizhou Medical University Guizhou P. R. China; ^3^ Division of Transplantation Immunology National Research Institute for Child Health and Development Tokyo Japan; ^4^ Department of Hepatobiliary Surgery Weifang People's Hospital Shandong P. R. China

**Keywords:** autoimmune disease, FOXP3, regulatory T cell, subpopulation, transplantation, tumor immunity

## Abstract

CD4^+^CD25^+^ regulatory T cells (Tregs), a subpopulation of naturally CD4^+^ T cells that characteristically express transcription factor Forkhead box P3 (FOXP3), play a pivotal role in the maintenance of immune homeostasis and the prevention of autoimmunity. With the development of biological technology, the understanding of plasticity and stability of Tregs has been further developed. Recent studies have suggested that human Tregs are functionally and phenotypically diverse. The functions and mechanisms of different phenotypes of Tregs in different disease settings, such as tumor microenvironment, autoimmune diseases, and transplantation, have gradually become hot spots of immunology research that arouse extensive attention. Among the complex functions, CD4^+^CD25^+^FOXP3^+^ Tregs possess a potent immunosuppressive capacity and can produce various cytokines, such as IL‐2, IL‐10, and TGF‐β, to regulate immune homeostasis. They can alleviate the progression of diseases by resisting inflammatory immune responses, whereas promoting the poor prognosis of diseases by helping cells evade immune surveillance or suppressing effector T cells activity. Therefore, methods for targeting Tregs to regulate their functions in the immune microenvironment, such as depleting them to strengthen tumor immunity or expanding them to treat immunological diseases, need to be developed. Here, we discuss that different subpopulations of Tregs are essential for the development of immunotherapeutic strategies involving Tregs in human diseases.

## Graphical abstract

Forkhead box P3 (FOXP3) regulatory T cells (Tregs) play a pivotal role in the maintenance of immune homeostasis and prevention of autoimmunity. Recent studies have suggested that human Tregs are functionally and phenotypically diverse. Here, we discuss that different Tregs subsets are essential for the development of immunotherapeutic strategies involving Tregs in human diseases.

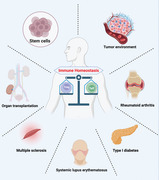

## 1 INTRODUCTION

Regulatory T cells (Tregs), the subset of T cells, are regarded as the central factor responsible for maintaining immunologic homeostasis and tolerance.^1^, ^2^, ^3^, ^4^, ^5^ In the original study, Gershon et al. identified a subset of T cells that had immunosuppressive functions.^6^ In the 1990s, Sakaguchi et al. described a unique subpopulation of CD4^+^ T cells in mice that were characterized by high expression of the IL‐2 receptor alpha chain (CD25).^7^ Since then, this group of CD4^+^CD25^+^ T cells has been named Tregs, which have been increasingly researched. In 2003, human and rodent CD4^+^CD25^+^ Tregs were found to specifically express the transcription factor Forkhead box P3 (FOXP3), which plays a crucial role in the differentiation and function of Tregs.^8^, ^9^, ^10^, ^11^ It is worth noting that FOXP3 protein is not only expressed in CD4^+^CD25^+^ Tregs, it can also be expressed in CD4^+^CD25^−^ T cells.^12^ The activation of peripheral CD4^+^CD25^−^ T cells can be transformed into CD4^+^CD25^+^ Tregs and induced to express FOXP3, which has similar immunoregulatory functions as natural CD4^+^CD25^+^FOXP3^+^ Tregs.^13^, ^14^ In addition, the IL‐7 receptor (CD127) is the surface marker of Tregs, which is downregulated in Tregs and is inversely correlated with the expression of FOXP3 in human peripheral blood.^15^ While Tregs are primarily known for their stable expression of FOXP3 and suppressive activity to prevent inflammation, deficiency of FOXP3 in both humans and mice results in the depletion of Tregs and leads to the development of severe systemic inflammatory diseases including immune dysregulation polyendocrinopathy enteropathy X‐linked (IPEX) syndrome,^16^, ^17^, ^18^ graft‐versus‐host disease (GVHD),^19^, ^20^, ^21^ solid organ transplantation,^22^, ^23^ type 1 diabetes (T1D),^24^, ^25^ systemic lupus erythematosus (SLE),^26^, ^27^ and multiple sclerosis (MS).^28^, ^29^ Moreover, Tregs depletion can evoke effective tumor immunity in the tumor microenvironment (TME).^30^, ^31^ These findings have stimulated advances in the clinical development of adoptive cell therapy (ACT) and targeted therapies of Tregs in the clinic, and early clinical trial results report excellent clinical safety and efficacy.^32^, ^33^, ^34^, ^35^, ^36^, ^37^, ^38^ Moreover, antigen‐specific Tregs^25^, ^39^, ^40^ and chimeric antigen receptors, and in genome editing Tregs‐based therapy^41^, ^42^, ^43^, ^44^, ^45^ exhibited potent benefits in autoimmunity and transplantation. Hence, the identification of specific Tregs as targets is of great clinical significance.

Among the various mechanisms of immunological self‐tolerance, immune suppression by endogenous CD4^+^CD25^+^FOXP3^+^ Tregs is essential and indispensable. With the help of a variety of biological detection techniques, including the most popular single‐cell sequencing,^46^, ^47^, ^48^ it is now well substantiated that numerous Tregs infiltrate various tumor tissues.^49^, ^50^, ^51^ Although controversy exists regarding the role of tumor‐infiltrating Tregs, the prevailing view is that they possess immunosuppressive functions, and their abundant presence is often associated with poor clinical prognosis.^52^ The removal of Tregs evokes effective antitumor immunity by abrogating immunological unresponsiveness, whereas the enrichment of specific Tregs subsets in patients with autoimmune diseases or transplant patients may help in suppressing the inflammatory response, which is conducive to the outcome.^53^

In this review, we focus on and select the representative markers of different subpopulations of Tregs that have been validated in samples from human sources and clinical trials, as well as our brief understanding of the functions and phenotypes of Tregs in human diseases. We also describe the relevant immune regulation mechanisms of these Tregs subsets and their potential as immunotherapeutic targets in the future.

## 2 TREG SUBSETS IN TUMOR IMMUNITY

### 2.1 Treg subsets in colorectal cancer

At present, increasing evidence suggests that FOXP3^+^ T cells in humans, including suppressive and nonsuppressive subpopulations, have heterogeneous phenotypes and functions.^4^, ^10^, ^49^, ^54^ The effect of abundant Tregs infiltrating tumor tissues such as colorectal cancers (CRCs) is controversial, and some studies have shown that the infiltration of FOXP3^+^ Tregs in tumor tissue is beneficial to the prognosis of patients.^55^, ^56^, ^57^, ^58^ According to previous research, FOXP3^+^CD4^+^ T cells circulating in human blood can be divided into three main groups based on FOXP3, CD25, and CD45RA: fraction I (Fr. I) CD45RA^+^CD25^low^/FOXP3^low^ naïve Tregs (nTregs), Fr. II CD45RA^−^CD25^high^/FOXP3^high^ effector Tregs (eTregs), and Fr. III CD45RA^−^CD25^low^/FOXP3^low^ cells, the majority of which are not Tregs.^54^ Based on this classification, Saito et al. demonstrated markedly increased numbers of Fr. II in both type A and B CRCs and that Fr. III cells were mainly increased in type B CRCs.^59^ Although Fr. II cells and Fr. III cells are both CD45RA^−^CD45RO^+^ effector‐type cells, only Fr. II cells from either type of CRCs tissue showed strong in vitro suppressive activity by highly expressing suppression‐related molecules, such as T‐cell immunoreceptor with Ig and ITIM domains (TIGIT) and cytotoxic T lymphocyte‐associated protein 4 (CTLA‐4). Fr. III nonsuppressive FOXP3^low^ cells were probably derived from non‐Tregs following activation with interleukin (IL)‐12 and transforming growth factor (TGF)‐β. Interestingly, type B CRCs showed significantly better recurrence‐free survival than type A CRCs. Thus, Fr. III nonsuppressive FOXP3^low^ cells could be a biomarker associated with better survival in CRCs. Furthermore, the strategy of increasing local FOXP3^low^ non‐Tregs may effectively inhibit the occurrence and development of CRCs.

**FIGURE 1:**
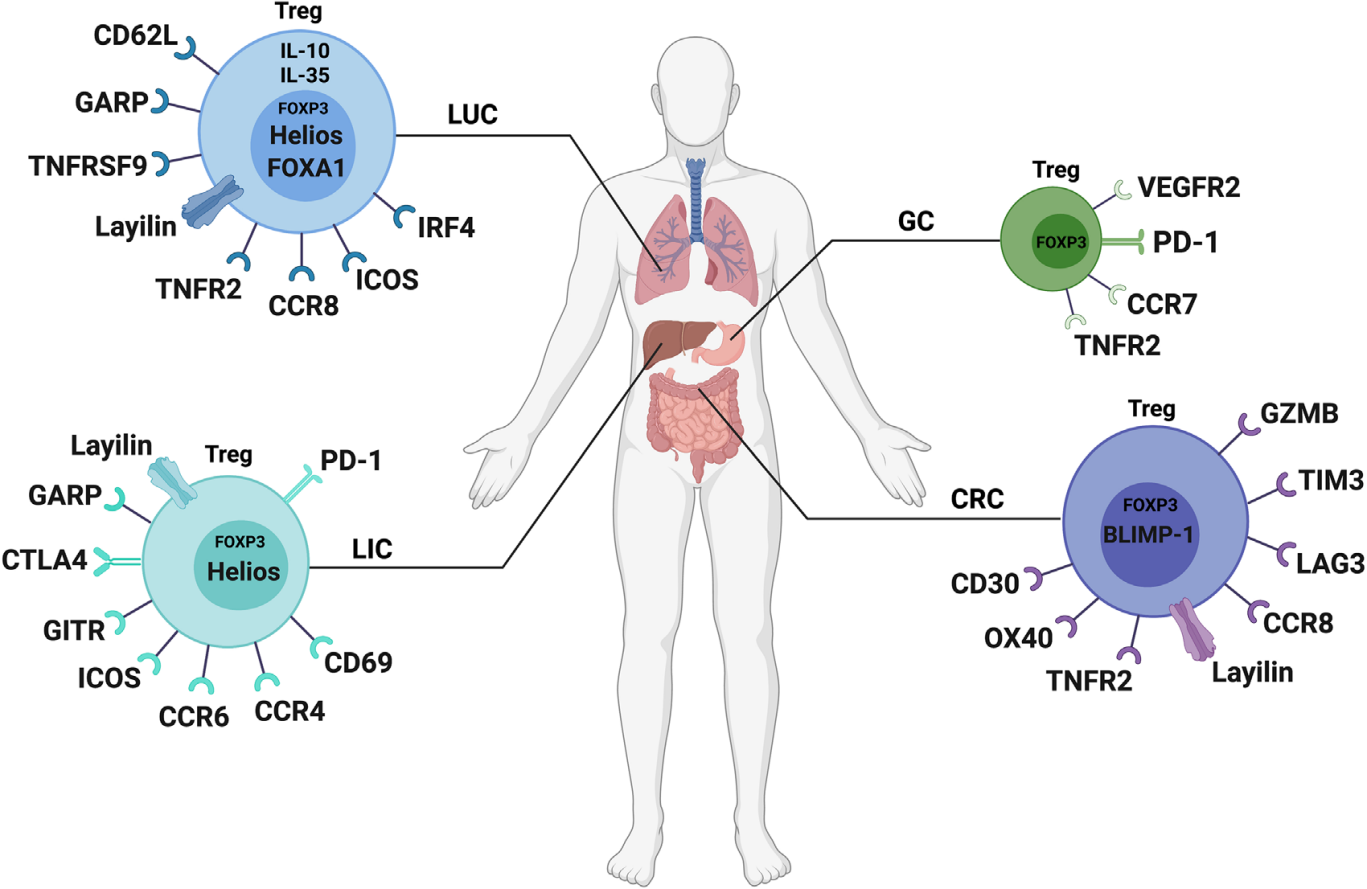
Different subpopulations of FOXP3^+^ Tregs in high‐incidence human cancers. This figure summarizes the subtypes of FOXP3^+^ Tregs described in colorectal cancer (CRC), lung cancer (LUC), liver cancer (LIC), and gastric cancer (GC)

A recent study reported that tumor‐infiltrating Tregs of CRC express granzyme B (GZMB) immediately after tumor resection, while there are almost no GZMB‐expressing Tregs in tumor‐associated lymph nodes and circulating lymphocytes.^60^ A number of studies have shown that GZMB‐expressing Tregs could induce the apoptosis of effector T cells^61^ and were also related to immune homeostasis, mediating tumor immunity,^62^, ^63^ viral infection, and transplant survival.^64^ Furthermore, the expression of granzymes was highly concentrated in T‐cell immunoglobulin‐ and mucin‐domain containing‐3 positive (TIM3^+^) Tregs, a subpopulation of Tregs that were enriched in the TME and presented increased suppressive capacity.^65^, ^66^, ^67^ GZMB^+^TIM3^+^ Tregs (Figure 1) were found to present higher cytolytic capacity toward autologous conventional T cells, which depended on GZMB rather than TIM3. Another study reported that CD4^+^CD25^+/high^FOXP3^+^ Tregs in peripheral blood mononuclear cells (PBMCs) of patients with CRCs could be divided into two subpopulations, consisting of lymphocyte activation gene 3 negative (LAG3^−^) TIM3^−^ Tregs and LAG3^+^TIM3^+^ Tregs (Figure 1), of which the latter accounted for more than half.^68^ LAG3^+^TIM3^+^ Tregs presented significantly higher expression of TGF‐β, IL‐10, and CTLA‐4 than LAG3^−^TIM3^−^ Tregs, similar to classical Tregs,^12^, ^69^, ^70^ which could suppress the expression of MHC‐II, CD80/CD86, and TNF‐α and increase the expression of IL‐10 in macrophages. These findings reported that LAG3^+^TIM3^+^ Tregs could lead to a worse prognosis and survival of CRCs patients.

CCR8 is a chemokine receptor predominantly expressed on Tregs in both mice^71^, ^72^ and humans.^30^, ^73^ As a subset of CD4^+^FOXP3^+^ Tregs, CCR8^+^ Tregs have been demonstrated to be a major driver of immunosuppression.^74^, ^75^, ^76^ Villarreal et al. found that tumor‐infiltrating CCR8^+^ Tregs are significantly upregulated in CRCs (Figure 1),^77^ similar to previous research in breast^78^ and lung cancer,^30^ and mAb therapy targeting CCR8 could obviously inhibit tumor growth and improve the prognosis in CRCs mouse models by increasing tumor‐specific T cells. Collectively, expressions of GZMB, LAG3 with TIM3, and CCR8 in FOXP3^+^ Tregs all strengthen their immunosuppressive function, and these markers may be potential new targets for immunotherapy of CRCs patients.

In addition, B lymphocyte‐induced maturation protein 1(BLIMP‐1), encoded by the PR domain zinc finger protein 1 (*PRDM1*) gene, is a multifunctional transcriptional regulator that has been previously found to be coexpressed with FOXP3 in murine Tregs.^79^ The research has shown that BLIMP‐1 expression defines eTregs as a distinct population of mature Tregs that produce IL‐10 and display an effector phenotype in mice. To understand the role of these cell populations in the immune regulation of human diseases, a pilot study has demonstrated that CRCs patients with low FOXP3^+^BLIMP‐1^+^ eTregs (Figure 1) infiltration in tumors are associated with a high risk of recurrence.^80^ Notably, not only prognostic value, the underlying mechanism of FOXP3^+^BLIMP‐1^+^ eTregs in CRCs patients remains to be verified.

Tumor necrosis factor receptor 2 (TNFR2), the receptor of TNF, has been found that is highly expressed in humans and mouse Tregs, especially on eTregs.^81^, ^82^, ^83^, ^84^, ^85^ Interestingly, the interaction of TNF with TNFR2 promotes suppressive function and phenotypical stability of Tregs.^86^, ^87^ Based on this interaction, Nie et al. have reported that blockade of TNFR2 signaling with an antibody could markedly reduce the tumor‐infiltrating TNFR2^+^ Tregs (Figure 1) in CRCs, whereas the number of tumor‐infiltrating interferon‐γ‐positive (IFN‐γ^+^)CD8^+^ cytotoxic T lymphocytes were significant increased.^88^ Moreover, the combination of TNFR2 blocking and immunotherapy showed a better efficacy, which will be more beneficial for the prognosis of CRCs patients.

OX40, also known as CD134 and TNFRSF4, is a member of the TNFR family which is identified as a negative regulator of FOXP3^+^ Tregs.^89^, ^90^, ^91^ Triggering OX40 with its ligand OX40L on the FOXP3^+^ Tregs abrogates its suppression of T‐cell proliferation and effector T cells (Teffs) function.^90^, ^91^, ^92^ Lam JH et al. have found that a high density of tumor‐infiltrating CD30^+^OX40^+^ Tregs (Figure 1) in CRCs patients is associated with improved prognosis, which may act as a diagnostic and prognostic biomarker in CRCs.^93^

### 2.2 Treg subsets in lung cancer

Lung cancer is known to be the leading cause of cancer‐related death worldwide.^94^ There are two subtypes of lung cancer: small‐cell lung cancer (SCLC: ∼15%) and non‐SCLC (NSCLC: ∼85%).^95^ Evidence from a wide range of sources suggests that high levels of Tregs are associated with metastasis, recurrence, and poor prognosis of lung cancer.^96^, ^97^, ^98^, ^99^, ^100^ Koyama et al. have shown that the proportion of CD62L^high^CD25^+^CD4^+^ Tregs (Figure 1) is significantly higher in extended‐stage disease SCLC, whereas there are more CD62L^low^CD4^+^ T cells in limited‐stage SCLC (LD‐SCLC).^100^ CD62L^low^CD4^+^ T cells that can produce IFN‐γ, IL‐4, and IL‐17 are considered to be immune Teffs. Th17 cells play an essential role in a variety of autoimmune diseases.^101^, ^102^, ^103^, ^104^, ^105^ These Teffs deviated to T‐helper type 17 (Th17) cells and were driven by IL‐23 secreted by monocyte‐derived DCs in LD‐SCLC. Unfortunately, CD62L^high^CD25^+^CD4^+^ Tregs can suppress cytokine production and inhibit the proliferation of Teffs. Therefore, a high Teffs to Tregs ratio could suppress the growth and metastasis of SCLC tumor cells. Based on this study, CD4^+^ T‐cell balance may be a helpful biomarker to assess immunologic responses, and increasing tumor‐reactive Teffs levels and depleting Tregs may be essential in establishing effective antitumor immunity.

Glycoprotein A repetitions predominant (GARP) has been proven to be highly expressed on the surface of Tregs.^106^ Previous studies have shown that GARP^+^ Tregs can secrete TGF‐β and possess a strong immunosuppressive function.^107^, ^108^, ^109^, ^110^, ^111^ Jin et al. demonstrated that GARP^+^ Tregs (Figure 1) were highly infiltrated in tumor tissues in the early stage of lung cancer and exerted immunosuppressive effects through the GARP‐TGF‐β pathway to inhibit Teffs.^112^ Therefore, tumor cells can escape immune surveillance and promote the occurrence and progression of tumors. Further research needs to confirm whether GARP is an important molecule of Tregs and a promising immunotherapy target.

A recent study reported increased numbers of FOXA1^+^ Tregs (Figure 1) in patients with lung cancer.^106^.The findings proved that FOXA1^+^ Tregs could inhibit the antitumor immunity of T cells and promote tumor growth by the IFN‐β‐PI3K‐Akt‐FOXA1 signaling pathway. Moreover, patients with more FOXA1^+^ Tregs showed more liver metastases and worse treatment responses. FOXA1 hepatocyte nuclear factor 3α is a transcription factor that is associated with the differentiation of embryonic stem cells, bile duct development, and cancer epigenetics.^113^, ^114^, ^115^, ^116^, ^117^ According to a previous study, Liu et al. demonstrated that FOXA1^+^ Tregs were induced by IFN‐β and possessed a suppressive function in experimental autoimmune encephalomyelitis (EAE) and MS models.^118^ This subset of Tregs may be a novel target of immunotherapy.

Helios (Ikzf2), a member of the Ikaros transcription factor family, was identified as a specific marker of FOXP3^+^ Tregs^119^, ^120^ and was shown to be involved in Tregs development and stability.^121^ FOXP3^+^Helios^+^ Tregs were present in thymus‐derived Tregs (tTregs),^122^ and FOXP3^+^Helios^−^ Tregs were present in peripherally‐induced Tregs (pTregs).^123^ Muto et al. have shown that FOXP3^+^ Helios^−^ Tregs (Figure 1) are apparently higher in NSCLC patients than in healthy controls.^124^ Interestingly, higher percentages of tumor‐infiltrated FOXP3^+^ Helios^−^ Tregs were seen in advanced‐stage NSCLC with poorer survival. Additionally, Guo et al. also found a subpopulation of Tregs named TNFRSF9^+^ Tregs (Figure 1), which were related to the poor prognosis of NSCLC.^125^ TNFRSF9 is tumor necrosis factor receptor superfamily member 9, also known as CD137 (4‐1BB), which is a surface marker that indicates antigen‐specific activation.^126^ Moreover, TNFRSF9^+^ Tregs are highly expressed immunosuppressive genes, such as *REL* ^127^ and *LAYN*,^47^ and may be a major component of functional tumor‐infiltrating Tregs in lung cancer. Although studies have shown that the high infiltration of FOXP3^+^Helios^−^ Tregs and TNFRSF9^+^ Tregs in tumors are not conducive to the prognosis of patients, the function and mechanisms of these subtypes of Tregs in tumor‐related immune suppression need to be further investigated.

Similar to the previous research, Yan et al. have found that TNFR2^+^ Tregs (Figure 1) could suppress the production of IFN‐γ by CD8^+^ T cells through expressed high levels of the CTLA‐4.^128^ As result, the high percentage of TNFR2^+^ Tregs in the peripheral blood of lung cancer patients was associated with lymphatic invasion, distant metastasis, more advanced clinical stage, and worse outcomes. Therefore, TNFR2 may prove to be a useful prognostic marker for lung cancer patients and as one of the best potential immune checkpoints on account of its critical role in TME.

Van Damme et al. and De Simone et al. have discerned that the high frequency of CCR8^+^ tumor‐infiltrating Tregs (Figure 1) plays a crucial suppressive function and is correlated with poor prognosis in patients with NSCLC.^30^, ^129^ The expression of CCR8 on the surface of tumor‐infiltrated Tregs may be induced by coregulation of NF‐κB and IFN regulatory factor 4 (IRF4).^130^, ^131^, ^132^ Remarkably, combination with anti‐CCR8 monotherapy could synergize the efficacy of anti‐PD‐1 therapy, which provides a beneficial treatment strategy for patients with NSCLC. Moreover, Alvisi et al. have demonstrated that the abundance of IRF4^+^ eTregs (Figure 1) correlated with poor prognosis in patients with NSCLC.^133^ The findings showed that high expression of IRF4 was present in CCR8^+^ICOS^+^ eTregs, which further contributed to the superior suppressive activity and worse disease‐free survival and overall survival of NSCLC patients. Therefore, anti‐IRF4 immunotherapy is needed to further research to benefit patients with NSCLC.

One of the ways that Tregs exert their suppression function is by secreting inhibitory cytokines including IL‐10, IL‐35, and TGF‐β.^134^, ^135^, ^136^, ^137^ Among them, IL‐35 appears to play a greater role in inhibitory receptor induction and restriction of central memory T‐ cell differentiation, while IL‐10 plays a greater role in regulating cytokine production and effector function. Sawant et al. found an abundance of IL‐10^+^ and IL‐35^+^ Tregs (Figure 1) in the TME of patients with NSCLC which cooperatively promoted exhaustion of BLIMP‐1‐dependent tumor‐infiltrating CD8^+^ T cells.^138^ The findings showed that IL‐10^+^ and IL‐35^+^ Tregs‐derived IL‐10 and IL‐35 collectively induced inhibitory receptors, such as TIM‐3 and LAG‐3 expression on intratumoral CD8^+^ T cells, which further resulted in T‐cell dysfunction and antitumor immunity failure. Therefore, the depletion by IL‐10^+^ and IL‐35^+^ Tregs may abrogate tumor‐immune evasive and resistance to immunotherapy of tumor cells.

### 2.3 Treg subsets in liver cancer

The liver has been recognized as an immune privilege organ.^139^, ^140^ Due to its complex immune regulation mechanism, current research is still unclear about its immune regulation. In recent years, there has been an increasing number of studies on the immune microenvironment of the liver at the single‐cell level. Zheng et al. used single‐cell RNA sequencing to reveal the landscape of infiltrating T cells in liver cancer.^47^ They showed that *LAYN* was preferentially upregulated in tumor‐infiltrated FOXP3^+^Helios^+^ Tregs. *LAYN* (Layilin), which was first reported in 1998, is a protein‐encoding gene located on chromosome11.^141^ *LAYN* has been defined as a Treg‐specific signature gene^142^ that is highly and specifically expressed in tumor‐infiltrating Tregs and correlates with poor prognosis in both NSCLC and CRCs patients.^30^ Similarly, high expression of tumor‐infiltrated LAYN^+^FOXP3^+^Helios^+^ Tregs (Figure 1), which possessed suppressive functions, was associated with tumor‐infiltrating exhausted CD8^+^ T cells and poor survival in liver cancer. In addition, Sun et al. also found that LAYN^+^ Tregs showed highly expressed *LAYN*, *TNFRSF9*, and inducible T‐cell costimulator (*ICOS*, *CD278*), but the proportion of this cell population in tumor‐infiltrating cells was very small, which was different from FOXP3^+^ Tregs.^48^ Hence, further studies are needed to generate mechanistic insights into the function of LAYN^+^ Tregs.

Similar to the Tregs subgroup mentioned above, Kalathil et al. defined GARP^+^/CTLA4^+^ Tregs as a subset of Tregs with immunosuppressive function in advanced liver cancer.^143^ The high frequency of GARP^+^/CTLA4^+^ Tregs (Figure 1) in patients with advanced hepatocellular carcinoma (HCC) may facilitate immune dysregulation, and the expression levels of GARP and CTLA were not associated with the viral infection. However, this finding suggests that depletion of these subpopulations of Tregs may be potential targets of immunotherapy.

In addition, Han et al. identified a new subset of tumor‐infiltrating CD4^+^CD69^+^ Tregs (Figure 1) that expressed neither CD25 nor FOXP3, but highly expressed membrane‐bound TGF‐β1 (mTGF‐β1), programmed cell death protein 1 (PD‐1, CD279), and CTLA‐4.^144^ These novel CD4^+^CD69^+^FOXP3^−^ Tregs accounted for the vast majority of tumor‐infiltrating Tregs compared with FOXP3^+^ Tregs and could suppress the CD4^+^ T‐cell response mainly through mTGF‐β1,^145^, ^146^ which was correlated with tumor progression. The underlying mechanisms of these tumor antigen‐induced CD4^+^CD69^+^FOXP3^−^ Tregs in HCC need further investigation for cancer immunotherapy.

Pedroza‐Gonzalez et al. have shown that the expression of glucocorticoid‐induced TNFR (GITR) and ICOS are upregulated in activated tumor‐infiltrating Tregs in patients with primary or metastatic liver cancer.^147^ GITR, also known as TNFRSF18, is a member of the TNF/nerve growth factor receptor family,^148^, ^149^, ^150^ and it is mainly expressed on CD4^+^CD25^+^ Tregs^151^ (Figure 1). The findings demonstrated that GITR ligation could abrogate the tumor‐infiltrating Tregs‐mediated suppression of effector T cells through treatment with soluble GITR ligand, which was consistent with previous research reports.^151^, ^152^, ^153^ Moreover, Tu et al. reported that ICOS^+^FOXP3^+^ Tregs (Figure 1) were significantly increased in the tumor tissues of patients with HCC.^154^ ICOS^+^ Tregs could produce a mass of IL‐10 and TGF‐β1,^155^ and the levels of tumor‐infiltrating ICOS^+^ Tregs were higher than those in tumor adjacent tissues in several cancers.^156^, ^157^, ^158^ Likewise, higher ICOS^+^ Tregs levels and ICOS^+^ Tregs/CD4^+^ T‐cell ratios indicated a worse prognosis in HCC. In addition, ICOS^+^ Tregs were concentrated in high chemokine (C‐C motif) ligand (CCL20)‐expressed areas. CCL20, the ligand targeting CCR6, has been demonstrated to be highly expressed in triggering receptors expressed on myeloid cell‐1 (TREM‐1)‐positive tumor‐associated macrophages (TAMs)^159^ in liver cancer. The findings also found that CCR6^+^ Tregs (Figure 1) accounted for the majority of intratumoral Tregs. Therefore, TREM‐1^+^ TAMs could recruit CCR6^+^ Tregs through producing CCL20, which induced CD8^+^ T‐cell exhaustion and was associated with poor prognosis in patients with HCC. In conclusion, CCL20 may be a potential target for immunotherapy.

A recent study has identified that CCR4^+^ Tregs (Figure 1) are the predominant type of Tregs in HBV^+^HCC.^160^ CCR4 is mainly expressed on Tregs and other T‐helper cells, which can mediate Tregs trafficking into the TME by interacting with its ligands, CCL22 and CCL17.^161^ The findings showed that the high frequency of CCR4^+^ Tregs exhibited potently immunosuppressive stem‐like specificity by upregulating transcription factor 1 (TCF1), PD‐1, and CTLA‐4 levels and secreting more IL‐10 and IL‐35. Moreover, targeting CCR4^+^ Tregs could alleviate sorafenib resistance and might improve the curative effect of immune checkpoint blockade (ICB) for patients with HBV^+^HCC.

What is noteworthy is that low glucose, or rather high‐lactic acid (LA), and hypoxic environments may impaired the survival and functions of effector T cells and are not conducive to antitumor immunotherapy.^162^, ^163^, ^164^ Kumagai et al. have reported that enhanced PD‐1 expression on eTregs (Figure 1) was observed in low‐glucose TME of HCC on account of eTregs actively absorbed LA through monocarboxylate transporter 1 (MCT1) and promoted nuclear factor of activated T cells‐1 (NFAT1) translocation into the nucleus.^165^ The deletion of MCT1, a transporter of LA,^166^, ^167^ plays a pivotal role for tumor‐infiltrating Tregs to uptake lactate to inhibit tumor growth and enhance immunotherapy response.^168^ In addition, NFAT1 positively regulates the expression of PD‐1 on T cells.^169^, ^170^, ^171^, ^172^ Therefore, molecular‐targeted immunotherapy against LA provides a novel insight for the clinic.

### 2.4 Treg subsets in gastric cancer

In recent years, increasing attention has been given to the application of ICBs, such as anti‐PD‐1 monoclonal antibodies (mAbs), in tumor immunity.^173^, ^174^ Some previous studies have shown that a fraction of Tregs expresses PD‐1.^175^, ^176^, ^177^, ^178^ However, the effects of anti‐PD‐1 mAbs on PD‐1^+^ Tregs in the tumor environment are currently poorly understood. Kamada et al. found that tumor‐infiltrating eTregs highly express PD‐1 and that the proliferation and immunosuppressive activity of PD‐1^+^ eTregs (Figure 1) could be reinforced by anti‐PD‐1 mAb therapy in patients with gastric cancer (GC).^179^ As a result, the patients treated with anti‐PD‐1 mAb aggravated the progression of the disease, which was consistent with the results of previous studies.^180^, ^181^ Therefore, the pros and cons of anti‐PD‐1 mAb therapy and its promotion of PD‐1^+^ eTregs suppressive function still need sufficient clinical data to verify.

Several studies have confirmed the limited efficacy of ICBs in the treatment of tumors,^182^, ^183^, ^184^, ^185^, ^186^, ^187^ and establishing more effective therapies is necessary. Vascular endothelial growth factor (VEGF) is secreted by a variety of tumors and accumulates abundantly in the TME, which mainly acts as an immunosuppressive factor.^188^ VEGF acts on the vascular endothelium through specific receptors, and VEGF receptor 2 (VEGFR2) is the most important signaling receptor.^189^, ^190^ In addition, VEGFR2 is selectively expressed by FOXP3^high^ CD4^+^ Tregs.^191^ Tada et al. revealed that VEGFR2^+^FOXP3^+^ eTregs (Figure 1) highly expressed Ki67 in GC tumor tissues.^192^ Ramucirumab (RAM), an anti‐VEGFR2 mAb,^193^, ^194^, ^195^ could reduce the frequency of VEGFR2^+^FOXP3^+^ eTregs. Furthermore, PD‐1 ligand (PD‐L1) expression and CD8^+^ T‐cell infiltration in tumor‐infiltrating lymphocytes were enhanced after treatment with RAM, which was beneficial to the killing of tumor cells. It can be seen that inhibiting the expression of VEGFR2 in Tregs may be a potentially beneficial treatment option.

T‐cell chemotaxis homing mediated by chemokine receptors plays a critical role in immune homeostasis.^196^ The chemokine receptor CCR7 has been proven to guide T‐cell chemotactic homing to tumor tissues.^197^ Furthermore, Mao et al. found that tumor‐infiltrating Tregs in GC expressed little CCR7 (Figure 1).^198^ Most tumor‐infiltrating Tregs exhibited a CD45RA^−^CCR7^−^ phenotype, which was induced by tumor‐derived TNF‐α and could inhibit the secretion of IFN‐γ and proliferation of effector CD8^+^ T cells. The high proportion of these subsets of Tregs in the tumor tissue was associated with tumor progression and poor prognosis. In the future, reversing the expression of CCR7 in Tregs may prevent tumor progression.

Qu et al. have also confirmed that TNFR2^+^ Tregs (Figure 1) preferentially accumulate in TME of GC, which express high levels of CTLA‐4 and CCR6 and possess strong suppressive activity by activating the TNF‐α/TNFR2 signaling pathway.^199^ In addition, the higher level of TNFR2^+^ Treg infiltration was correlated to a poorer prognosis in GC patients. Based on the results, targeting TNFR2^+^ Tregs may be an immunotherapeutic strategy for GC.

### 2.5 Treg subsets in other cancers

**FIGURE 2:**
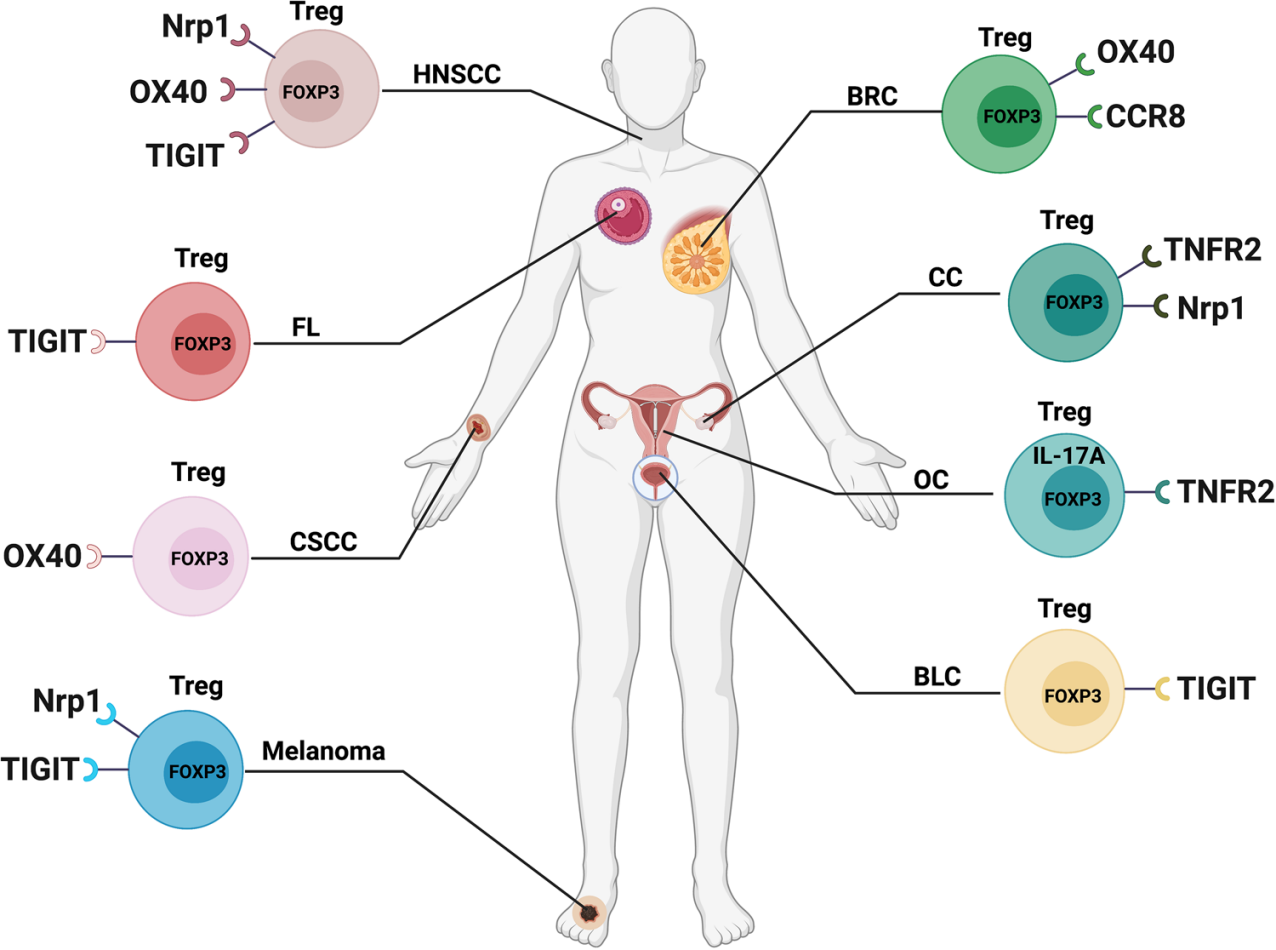
Different subpopulations of FOXP3^+^ Tregs in other cancers. This figure summarizes the subtypes of FOXP3^+^ Tregs described in neck squamous cell carcinoma (HNSCC), follicular lymphoma (FL), cutaneous squamous cell carcinoma (CSCC), melanoma, breast cancer (BRC), cervical cancer (CC), ovarian cancer (OC), bladder cancer (BLC)

Many different subtypes of Tregs have also been found in other types of tumors. CCR8 has been identified to be upregulated in intratumoral Tregs in a variety of tumors.^30^, ^78^ A recent study on breast cancer showed that CCR8^+^ Tregs (Figure 2), a subset of peripheral blood Fr. II, could be recruited to human breast tumors through the CCL1‐CCR8 axis.^200^ Intratumoral CCR8^+^ Tregs highly expressed surface‐activated markers such as CTLA‐4, CD39, and PD‐1. Activated CCR8^+^ Tregs have the potential for immunosuppression and are significantly related to the clinical outcome of patients with breast cancer.

Neuropilin‐1 (Nrp1), a cell surface glycoprotein of the semaphorin III receptor, was reported to be an exclusive surface marker of Tregs and is associated with suppressive activity.^201^, ^202^ Indeed, the expression of Nrp1 on human Tregs has always been controversial, with some suggesting that peripheral human Tregs do not express Nrp1 while others suggest that Nrp1 is expressed on a subset of CD8^+^ TIL in human NSCLC.^203^, ^204^ In many subsequent studies, Battaglia et al. have shown that depletion of Nrp1^+^ Tregs (Figure 2) in tumor‐draining lymph nodes is directly related to a favorable response to chemoradiotherapy in cervical cancer.^205^ Overacre‐Delgoffe et al. have found that a high proportion of human Tregs expressed Nrp1 in melanoma and neck squamous cell carcinoma (HNSCC) and peripheral blood lymphocyte (PBL) Tregs from these cancer patients also possessed a clear population of Nrp1^+^ Tregs in contrast to healthy donor PBL Tregs.^206^ Interestingly, Nrp1 expression in intratumoral Tregs appeared to correlate with poor prognosis in both melanoma and HNSCC. The findings also suggested that targeting Nrp1^+^ intratumoral Tregs with an Nrp1 mAb may be therapeutic. Furthermore, Bell et al. have also found that OX40 (Figure 2) is particularly expressed on the surface of Tregs of HNSCC patients.^207^ The anti‐OX40 clinical trial is expected to be conducted in patients with advanced cancers and yield good clinical benefits.

Not only that, but several studies have also found that OX40 (Figure 2) is predominantly expressed on the tumor‐infiltrated FOXP3^+^ Tregs of cutaneous squamous cell carcinoma^208^ and peripheral FOXP3^+^ Tregs of patients with breast cancer.^209^ The application of anti‐OX40 could promote tumor‐infiltrated CD4^+^ T‐cell proliferation and reduce tumor metastasis,^208^ which provides a therapeutic target for tumors.

Govindaraj et al. have revealed that TNFR2^+^ Tregs (Figure 2) are presented at high levels within the ascites of ovarian cancer, which are more suppressive compared to peripheral blood TNFR2^+^ Tregs.^210^ TNFR2^+^ Tregs mainly expressed high levels of CD39, CD73, TGF‐β, and GARP in the cell surface to increase their suppressive capacity, which further inhibited the production of IFN‐γ by Teffs. Moreover, Zhang et al. demonstrated that TNFR2^+^ Tregs (Figure 2) infiltrated both peripheral and tumors in patients with cervical intraepithelial neoplasia and cervical cancer, and the proportion of TNFR2^+^ Tregs was found to be associated with the clinical stages of cervical cancer.^211^ Therefore, TNFR2^+^ Tregs may be a potential immunotherapy target to improve survival for patients with ovarian cancer and cervical cancer.

Th17 cells have considerable plasticity in autoimmune diseases and other chronic inflammatory diseases and mainly produce IL‐17.^212^, ^213^, ^214^, ^215^ Th17 cells and Tregs are indispensable in maintaining immune homeostasis, and Th17‐Treg imbalance is related to inflammatory immunosuppression in cancer. Downs‐Canner et al. revealed that Th17 cells presented a novel source of tumor‐induced FOXP3^+^ Tregs in tumor‐bearing mouse models.^216^ It is generally known that TGF‐β is essential in producing pTregs, iTregs, and some TH17 cells,^217^, ^218^ and the above findings have demonstrated that Th17 (IL‐17A^+^FOXP3^−^) cells progressively transdifferentiate into IL‐17A^+^FOXP3^+^ and ex‐Th17 IL‐17A^−^FOXP3^+^ T cells promoted by TGF‐β during tumor progression, which are subpopulations of suppressive Tregs (Figure 2). In contrast, when FOXP3^+^ Tregs are exposed to IL‐6 with or without IL‐1β and IL‐23, FOXP3 becomes downregulated in favor of expressing Th17 genes including *IL‐17*, *IL‐22*, IL‐23‐receptor (*IL‐23R*), and retinoid‐related orphan receptor‐γt (*RORγt*).^219^ Based on this report, inhibiting the conversion of Th17 cells into Tregs may serve as a valuable targeting strategy in tumor immunotherapy.

As mentioned in the previous description, TIGIT is an immunosuppressive‐related immunoreceptor expressed on eTregs (Fr. II), which can facilitate Tregs development.^220^, ^221^, ^222^ DNAM‐1 (DNAX accessory molecule‐1, CD226),^223^ a competitive costimulatory counter‐receptor of TIGIT,^220^, ^224^, ^225^ enhances T‐cell activation,^223^, ^226^ while TIGIT can inhibit T‐cell responses through T cell‐intrinsic inhibitory functions.^227^ Fourcade et al. found that tumor‐infiltrating Tregs express high‐level TIGIT and low‐level CD226 in melanoma.^228^ TIGIT^+^ Tregs (Figure 2) showed suppression and stable characteristics as well as a large amount of enrichment in tumors. The research indicated that a high proportion of TIGIT^+^ Tregs and a high TIGIT/CD226 ratio in Tregs in the tumor immune microenvironment were associated with poor clinical prognosis. Recently, Wu et al. have found that the frequency of TIGIT^+^ Tregs was significantly increased in patients with bladder cancer by single‐cell sequencing.^229^ Interestingly, the TIGIT^+^ Tregs also highly expressed IL‐32 which further promoted the migration and invasion of tumor cells. As a result, targeting TIGIT to suppress the metastasis of tumor may be a novel insight for patients with bladder cancer. In addition, Yang et al. have demonstrated that abundantly tumor‐infiltrating TIGIT^+^ Tregs show enhanced suppressive capacity by inhibiting the activation and proliferation of CD8^+^ T cells in patients with follicular lymphoma.^230^ The findings also identified several other types of TIGIT^+^ T cell subsets, including exhausted TIGIT^+^ non‐Tregs/follicular helper T cells (Tfhs) and effector TIGIT^+^ T cells. In conclusion, increased numbers of TIGIT^+^ T cells were correlated with inferior outcomes and poor survival and the blockade of TIGIT signaling may have therapeutic potential for patients with follicular lymphoma. Moreover, another study reported that the expression of TIGIT (Figure 2) elevated in the PBMCs and tumor tissues of patients with HNSCC.^231^ TIGIT could activate its ligand CD155,^232^ and the activation of TIGIT/CD155 signaling was associated with the pathologic grade and lymph node metastasis of HNSCC. Consistent with previous studies, targeting TIGIT/CD155 signaling could reduce the secretion of TGF‐β and may be a potential therapeutic strategy for HNSCC patients.

## 3 TREG SUBSETS IN AUTOIMMUNE DISEASE

### 3.1 Treg subsets in systemic lupus erythematosus

**FIGURE 3:**
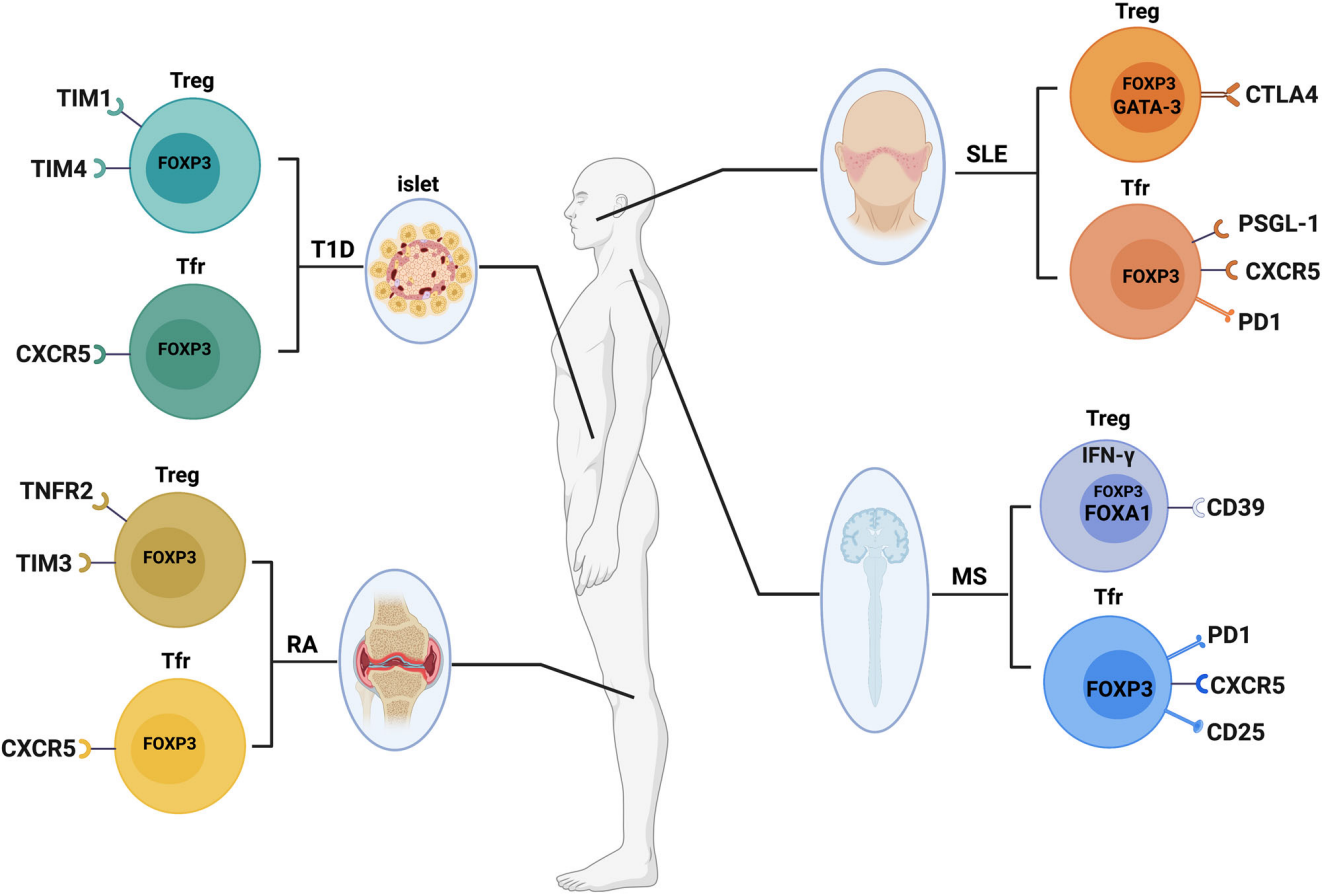
Different subpopulations of FOXP3^+^ Tregs in autoimmune diseases. This figure summarizes the subtypes of FOXP3^+^ Tregs described in autoimmune diseases including systemic lupus erythematosus (SLE); multiple sclerosis (MS), type 1 diabetes (T1D), and rheumatoid arthritis (RA)

SLE, a chronic autoimmune systemic disease with extremely varied clinical manifestations and complex pathogenesis, has been characterized by the breakdown of immunological tolerance and antinuclear antibody production.^233^ Some studies have shown that the suppressive function of Tregs is weakened in patients with active SLE, but the specific mechanisms remain unknown.^234^, ^235^ It was recently reported that IL‐21, a proinflammatory cytokine in SLE, could suppress FOXP3^+^ Tregs differentiation and suppressive activity by inhibiting CTLA‐4 and GATA‐3 expression.^236^ GATA‐3 is a transcription factor that mediates the expression of FOXP3^237^ and is essential for Tregs development and function.^238^ CTLA‐4 is associated with Tregs activation. Interestingly, in contrast to IL‐21, IL‐2 and TGF‐β promoted the expression of CTLA‐4^+^ Tregs and GATA‐3^+^ Tregs in SLE (Figure 3). Therefore, inhibiting the production of IL‐21 from the source can restore the suppressive function of CTLA‐4^+^ Tregs and GATA‐3^+^ Tregs, which may be beneficial to the treatment of SLE patients.

Follicular regulatory T cells (Tfrs), one of the subsets of Tregs that specifically regulate the function of Tfhs through expressing CXCR5 and FOXP3, mainly suppress the activation of Tfhs in germinal centers to maintain immune homeostasis.^239^, ^240^, ^241^ The dysregulation of Tfrs may eventually contribute to the progression of autoimmune diseases.^242^, ^243^, ^244^ Liu et al. have demonstrated an elevated level of CD4^+^CXCR5^+^FOXP3^+^ Tfrs (Figure 3) in peripheral blood of SLE patients.^244^ The high proportion of Tfrs could suppress Tfhs function and the frequency of Tfrs was positively associated with autoantibodies (AAbs) and clinical severity of SLE patients, which might be a response to the enhanced humoral immunity and provided novel insight during SLE pathogenesis. In subsequent studies, researchers have found that IL‐2 plays a pivotal role in maintaining the suppression function of Tfrs.^245^, ^246^, ^247^ When serum IL‐2 levels were reduced in patients with SLE, the proportion of CD4^+^CXCR5^+^FOXP3^–^PD‐1^high^ Tfhs was increased, whereas the proportion of CD4^+^CXCR5^+^CD45RA^–^FOXP3^high^ Tfrs was decreased.^245^ However, stimulation with an exogenous low dose of IL‐2 could restore the function of Tfrs and converse Tfhs to Tfrs by regulation of FOXP3 and BCL6 through histone modification. Similarly, high expression of PD‐1 was explored on Tfrs (Figure 3) with dysfunction of suppressing Tfhs proliferation and activation in patients with SLE due to IL‐2 deficiency, which could also be rescued by low‐dose IL‐2 treatment.^246^ As result, IL‐2 treatment may provide potential therapeutic benefits for SLE.

A recent study has identified that P‐selectin glycoprotein ligand‐1 (PSGL‐1), an adhesion molecule that can be expressed by T cells,^248^, ^249^ is highly expressed on Tfrs (Figure 3) in patients with SLE.^250^ PSGL‐1 interaction with selectin impaired the suppression function of Tfrs and contributed to the pathogenesis of SLE via inhibition of the TGF‐β pathway and reduced expression of FOXP3. Moreover, the circulating P‐selectin derived from platelets was activated and correlated with disease severity in the patients with SLE. Blocking P‐selectin may improve inflammation and as a potential therapeutic target for SLE.

### 3.2 Treg subsets in MS

In 1992, MS was described as an autoimmune disease caused by pathogenic T cells specific for myelin‐Ags in the central nervous system.^251^ At present, research on the function of Tregs in MS patients is still controversial.^252^, ^253^ In addition to Th1 cells, studies have reported that IL‐17 is highly expressed in MS patients, so it could be speculated that Th17 cells play a key role in mediating autoimmune inflammation.^104^, ^254^ Moreover, it has been reported that human FOXP3^+^ Tregs can secrete IL‐17.^255^ Subsequently, however, Fletcher et al. demonstrated that FOXP3^+^CD39^+^ Tregs (Figure 3) could suppress the production of IL‐17 by Th17 cells, while FOXP3^+^CD39^−^ Tregs could produce IL‐17 in MS patients.^256^ Unfortunately, the proportion of FOXP3^+^CD39^+^ Tregs was reduced, and their function was impaired. CD39 is an ectonucleotidase that can hydrolyze ATP into AMP, and the hydrolysis of ATP is considered to be an important mechanism of immunoregulation.^257^ As a result, injection of FOXP3^+^CD39^+^ Tregs and exclusion of FOXP3^+^CD39^−^ Tregs may be a potential immunotherapy strategy for MS.

In the previous description, we mentioned that FOXA1^+^ Tregs (Figure 3) were found in lung cancer patients, which were reported earlier in EAE and MS models.^118^ The findings showed that FOXA1 could bind to PD‐L1, which was essential for FOXA1^+^ Tregs to kill activated T cells. More importantly, in addition to the IFN‐α/β receptor signaling inherent in T cells, the development of FOXA1^+^ Tregs could also be induced by IFN‐β. Therefore, clinical treatment with IFN‐β to induce more suppressive FOXA1^+^ Tregs may generate favorable outcomes for MS patients.

Similarly, Dominguez‐Villar et al. found that IFN‐γ^+^FOXP3^+^ Tregs (Figure 3) were significantly more abundant than IFN‐γ^−^FOXP3^+^ Tregs in MS.^258^ IL‐12, a proinflammatory cytokine that is highly expressed in individuals with MS,^259^ could stimulate the secretion of IFN‐γ by Tregs. The findings indicated that the suppressive activity of IFN‐γ^+^FOXP3^+^ Tregs was inhibited through the secretion of IFN‐γ stimulated by IL‐12. Prospective studies may focus on the frequency of IFN‐γ^+^FOXP3^+^ Tregs at risk for developing MS.

Recently, Haque et al. have evaluated the frequencies of circulating CXCR5^+^PD‐1^+^ Tfhs and CXCR5^+^PD‐1^+^ FOXP3^+^CD25^+^ Tfrs (Figure 3) in patients with MS.^260^ It was proven that the frequency of circulating CXCR5^+^PD‐1^+^ Tfhs was increased in MS patients, whereas the frequency of circulating CXCR5^+^PD‐1^+^ FOXP3^+^CD25^+^ Tfrs was significantly decreased. Consistent with previous research, a high proportion of circulating CXCR5^+^PD‐1^+^ Tfhs secreted abundance of IL‐21,^261^ which contributed to the production of pathogenic autoantibody.^262^, ^263^, ^264^ Correspondingly, a lower frequency of circulating Tfrs was correlated to the reduction of IL‐10 which might increase the severity of MS.^265^, ^266^, ^267^ The findings suggested that improved IL‐10 secretion by circulating Tfrs may serve as a potential therapeutic target for patients with MS.

### 3.3 Treg subsets in rheumatoid arthritis

Rheumatoid arthritis (RA), one of the common types of chronic and pharmacologically complex systemic autoimmune disease observed in the elderly population,^242^, ^268^, ^269^, ^270^ is characterized by the presence of AAbs such as rheumatoid factor (RF) and anti‐cyclic citrullinated peptide antibody.^271^, ^272^ Liu et al. have found that the frequency of circulating CD4^+^CXCR5^+^FOXP3^+^ Tfrs (Figure 3) and the ratio of Tfrs/Tfhs were significantly increased in patients with stable RA than in patients with active RA or healthy people.^242^ The circulating CD4^+^CXCR5^+^FOXP3^+^ Tfrs with enhanced suppressive function could alleviate autoimmunity in RA patients by reducing IgG and IgM levels, and the proportion of Tfrs was negatively correlated with the disease severity, which provided a novel insight into RA pathogenesis.

Santinon et al. have also demonstrated that the suppressive TNFR2^+^ Tregs (Figure 3) play a key role in patients with RA.^273^ They confirmed that TNF treatment could promote the proliferation of TNFR2^+^ Tregs and ameliorate inflammation by activation of TNF–TNFR2 signaling^274^, ^275^ and enhance the expression of CD25. Targeting TNFR2 signaling may be a potential therapeutic strategy in RA.

Given that TIM3^+^FOXP3^+^ Tregs possess potent suppression of proinflammatory responses, Sun et al. have examined the frequency and function of TIM3^+^FOXP3^+^ Tregs (Figure 3) in patients with RA.^276^ They demonstrated the proportion of TIM3^+^FOXP3^+^ Tregs, which could potently suppress IFN‐γ and TNF‐α inflammation from Teffs by producing high IL‐10 and TGF‐β, was significantly decreased in patients with RA. As result, the frequency of TIM3^+^FOXP3^+^ Tregs was negatively associated with the pathogenesis of RA, which might be an immunotherapeutic target for patients with RA.

### 3.4 Treg subsets in type 1 diabetes

T1D, a chronic autoimmune disorder, is characterized by insulin deficiency and resultant hyperglycaemia.^277^, ^278^, ^279^ The complex interactions between the pancreatic β‐cell and innate and adaptive immune systems lead to the progression of T1D.^280^ In humans, Tfrs are identified that can inhibit the production of AAbs and restore glucose tolerance.^239^, ^281^, ^282^ Vecchione et al. have reported that the frequency of CXCR5^−^FOXP3^+^ Tregs was higher in AAb^+^ patients than in AAb^−^ patients, which may be caused by active autoimmunity and metabolic dysfunction in AAb^+^ patients.^279^ Furthermore, they also validated that CXCR5^+^FOXP3^+^ Tfrs (Figure 3), which were needed to maintain peripheral tolerance by regulating diabetogenic Tfhs and B cells, were reduced in spleen and pancreatic lymph node of patients with T1D. Therefore, Tfrs ACT may be a potential therapeutic modality to treat T1D.

The TIM family comprises TIM1, TIM3, and TIM4, and there is little known about the expression of TIM1 and TIM‐4 on Tregs. Guo et al. have evaluated the frequencies of circulating TIM1^+^ Tregs and TMI4^+^ Tregs (Figure 3) in patients with TID.^283^ Their study revealed that TIM1^+^ Tregs and TMI4^+^ Tregs were significantly decreased in PBMCs of patients with T1D as compared with healthy people. Inhibition of TIM1 could reduce FOXP3 expression and inhibit Tregs development,^284^ while TIM4 regulated the activation of naive T cells and proliferation of activated T cells.^285^, ^286^ However, the specific functions and potential mechanisms of TIM1^+^ Tregs and TMI4^+^ Tregs in patients with T1D may need to be further explored.

## 4 TREG SUBSETS IN TRANSPLANTATION

### 4.1 Treg subsets in

**FIGURE 4:**
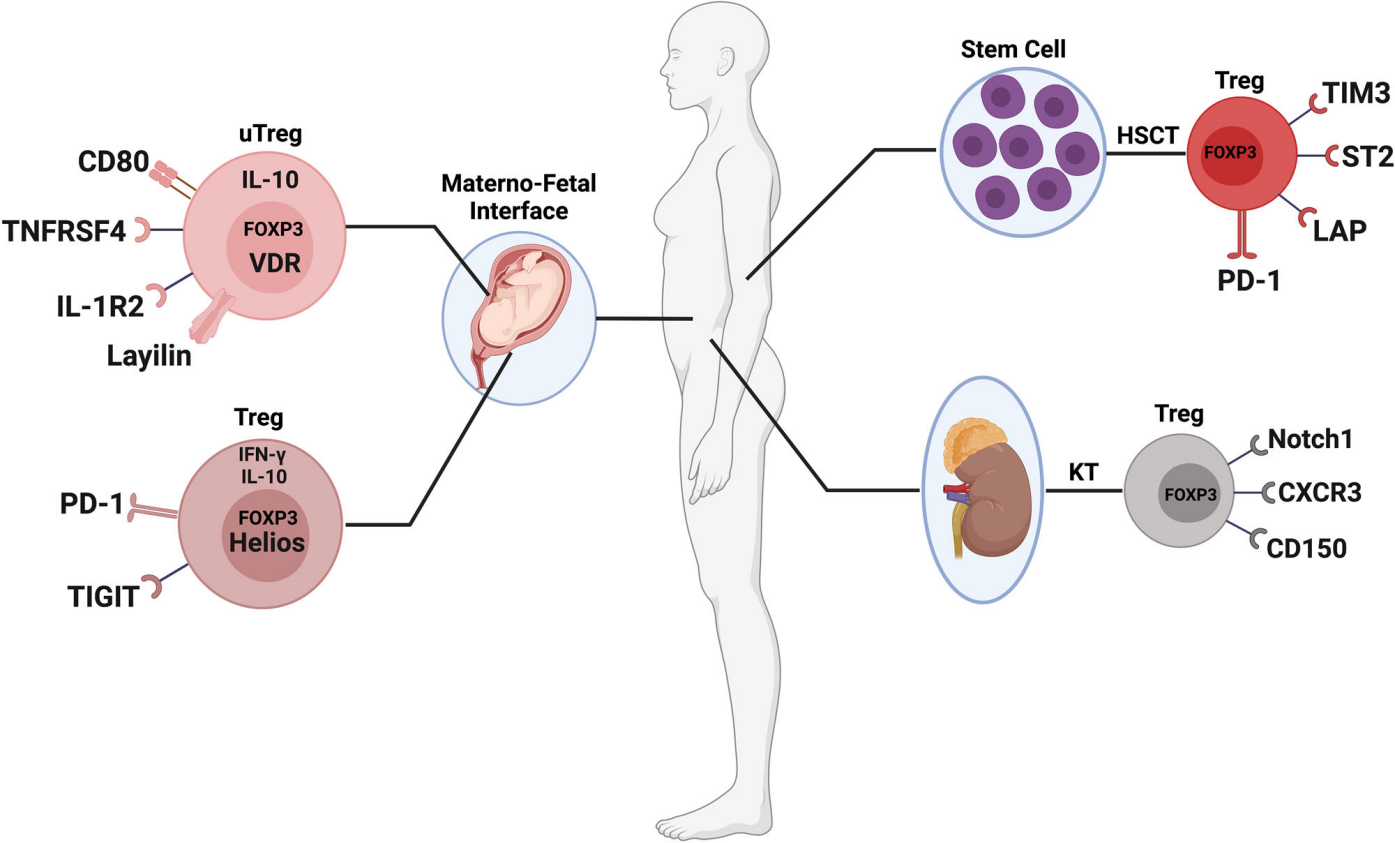
Different subpopulations of FOXP3^+^ Tregs in transplantation and pregnancy. This figure summarizes the subtypes of FOXP3^+^ Tregs described in kidney transplantation (KT), hematopoietic stem cells transplantation (HSCT), and materno‐fetal interface

Although the current improvement in surgical techniques and the application of immunosuppression have improved survival after organ transplantation, immune‐mediated injury is still a critical cause of allograft failure.^287^, ^288^ Notch signaling plays a significant role in immune cell fate determination and differentiation.^289^ Notch‐1, a Notch receptor of the Notch family, is pivotal for regulating fate decisions in the T‐cell lineage.^290^ More recently, Magee et al. found that Notch‐1 was highly expressed in FOXP3^+^ Tregs and that the proportion of Notch‐1^+^ Tregs (Figure 4) was significantly higher in patients with a kidney transplant than in healthy controls.^291^ Furthermore, the results showed that inhibition of Notch‐1 could reduce the frequency and function of effector T cells, whereas the survival, proliferation, and suppressive function of Tregs were enhanced. In agreement with a previous study,^292^ treatment targeting Notch‐1^+^ Tregs may improve allograft tolerance and prolong the survival of solid organ transplantation in humans.

In addition, Hoerning et al. confirmed that the expression of CXCR3 on human CD4^+^FOXP3^+^ Tregs was associated with better renal transplant function (Figure 4).^293^ It has been reported that CXCR3 promotes Tregs recruitment and Teffs interactions, which limits autoimmune‐mediated tissue damage in EAE.^294^ The CXCR3^+^FOXP3^+^ Tregs also highly expressed GARP, indicating that this subset of Tregs possessed a strong immunosuppressive function. Interestingly, treatment with tacrolimus did not affect the expression of CXCR3 on the surface of Tregs. CXCR3^+^FOXP3^+^ Tregs showed the capacity to translocate to the site of inflammation and could control renal allograft rejection.

### 4.2 Treg subsets in graft‐versus‐host disease

Acute GVHD is a very common complication in hematopoietic stem cells transplantation (HSCT).^295^ Trzonkowski et al. first described a method for the adoptive transfer of CD4^+^CD25^+^CD127^−^ Tregs expanded in vitro for the treatment of patients with acute and chronic GVHD, which could improve their condition.^296^ Subsequently, several HSCT clinical phase I and phase II studies were carried out in human leukocyte antigen (HLA)–haploidentical^297^, ^298^ or umbilical cord blood^299^ transplantation setting and using HLA‐matched donors,^300^ which further confirmed the stability of the immunosuppressive function of Tregs expanded in vitro and the feasibility of preventing GVHD. In addition, some more detailed induction strategies and subtypes of Tregs have been proved to have stronger mechanisms of suppressive function. Among them, two clinical studies have demonstrated that low‐dose IL‐2 can better induce the expansion of functional Tregs and ameliorate the manifestations of chronic GVHD (cGVHD).^301^, ^302^ Notably, Asano et al. have demonstrated that low doses of IL‐2 enhance PD‐1 expression of central memory Tregs in patients with cGVHD, suggesting that the PD‐1 pathway is a critical homeostasis and tolerance regulator for Tregs.^178^ Ulbar et al. have used an automated, clinical‐grade protocol to expand Tregs in vitro.^303^ The expression levels of IL‐10, GZMB, and IL‐35 of these expanded Tregs were significantly increased, and could protect mice from GVHD. Among them, the subtype of TIM3^+^ Tregs (Figure 4) exhibited a highly immunosuppressive function. We hope these findings may facilitate the development of therapeutic strategies that promote immune tolerance and reverse symptoms of GVHD in humans.

IL‐33 and its receptor suppression of tumorigenicity‐2 (ST2) have been reported to be associated with Tregs immunobiology, and ST2^+^FOXP3^+^ Tregs (Figure 4) could be augmented following administration of IL‐33.^304^, ^305^, ^306^ Consequently, Matta et al. demonstrated that IL‐33 mediated the expansion of ST2^+^FOXP3^+^ Tregs via activation of p38 MAPK signaling after HSCT, which could protect against acute GVHD.^307^ In xenogeneic GVHD (xGVHD), Wang et al. have demonstrated that latency‐associated peptide (LAP) can be used as a unique cell‐surface marker to distinguish bona fide Tregs from activated FOXP3^+^ and FOXP3^−^ non‐Tregs, which is beneficial to improve the purity of Tregs expanded in vitro.^308^ The subset of LAP^+^FOXP3^+^ Tregs (Figure 4) possessed a fully suppressive function and adoptive transfer of LAP^+^ Tregs could delay the development of xGVHD. It may be important to testify their safety and stability in the future.

The bone marrow niche is important for the potential of HSCs to maintain multifunctionality, such as quiescent status, multidirectional differentiation, and self‐renewal, in which the adenosine signaling pathway plays a pivotal role.^309^, ^310^ It was worth noting that CD150^+^ Tregs (Figure 4) could secrete adenosine to maintain the quiescent status of HSCs, which activated the AMPK pathway to promote energy metabolism to inhibit the GVHD and intestinal cell apoptosis secondary to HSCT. Their results provided potential feasibility of inducing the expression of CD150^+^ Tregs as the therapeutic strategy to prevent GVHD for patients after HSCT.

## 5 TREG SUBSETS AT MATERNO‐FETAL INTERFACE

During pregnancy, maternal Tregs are considered to be very important in establishing maternal‐fetal immune tolerance and are indispensable for successful embryo implantation and pregnancy outcome.^311^, ^312^ A large number of Tregs are enriched in the gestational uterus, maternal periphery, and maternal‐fetal interface in normal human pregnancy.^313^, ^314^, ^315^, ^316^ To more thoroughly understand the immune regulation mechanism of the maternal‐fetal interface, one of the most interesting yet elusive tissue sites for Tregs function in humans, Wienke et al. have used uterine Tregs (uTregs) (Figure 4) from the maternal‐fetal interface for transcriptome profile and functional adaptation analysis, which comprehensively reveals the gene expression profile of uTregs as bona fide suppressive Tregs.^317^ Moreover, uTregs also exhibited a special phenotype similar to tumor‐infiltrating Tregs, which highly expressed IL1R2, LAYN, CD80, TNFRSF4, etc. Although these findings deepen our understanding of Tregs at the maternal‐fetal interface and show great clinical application potential, the clinical application of these phenotypes still needs more trials to verify.

It is worth noting that Salvany‐Celades et al. have defined three subtypes of decidual CD4^+^ Tregs that possess regulatory functions and suppress T‐cell responses during human pregnancy including CD25^high^HELIOS^+^FOXP3^+^, PD‐1^high^IL‐10^+^, and TIGIT^+^FOXP3^low^ Tregs^318^ (Figure 4). PD‐1^high^IL‐10^+^ and TIGIT^+^FOXP3^low^ Tregs could significantly inhibit CD4^+^ T cells proliferation while CD25^high^HELIOS^+^FOXP3^+^ Tregs could simultaneously inhibit the proliferation of CD4^+^ and CD8^+^ Teffs and affect their production of certain cytokines. This research provides strong evidence that maintaining maternal‐fetal immune homeostasis requires multiple types of Tregs to perform important functions.

## 6 CONCLUSIONS AND PERSPECTIVES

An increasing amount of evidence indicates that Tregs play significant roles in various immunological and inflammatory diseases. In this review, the functions and brief mechanisms of different subpopulations of FOXP3^+^ Tregs in diverse immune microenvironments are discussed (Tables 1, 2). In general, we consider that most subtypes of Tregs, such as TNFR2^+^, LAG3^+^, TIM3^+^, CTLA‐4^+^, which possess potent suppressive functions, mainly promote tumor progression by inhibiting the proliferation and activity of effector CD4^+^ T cells and tumor‐killing CD8^+^ T cells in the TME of several cancers. As a result, the strategy of depleting the suppressive Tregs can prevent tumor cells from evading immune surveillance, which may be a promising target for tumor treatment. With the rapid development of biological technology, however, researchers have also identified some subtypes of Tregs that are highly infiltrated in tumors are associated with beneficial prognosis of patients. The high frequencies of tumor‐infiltrating CD30^+^OX40^+^ Tregs and BLIMP‐1^+^FOXP3^+^ Tregs exhibit a favorable aspect, especially to reduce the risk of recurrence, for patients with CRCs. Meanwhile, it is far from enough to understand the function of these subtypes of Tregs in the TME, and more in‐depth mechanistic studies are also needed to determine whether in vitro expansion and reinfusion of these Tregs can help patients inhibit tumor progression in the future. Another noteworthy situation is that the efficacy of targeted therapy is controversial. For example, anti‐PD‐1 mAb therapy is not only ineffective, but aggravates tumor progression in some patients with GC. Therefore, the clinical efficacy and safety of targeted therapies still need large‐scale clinical trials to be further clarified. In the treatment of patients with autoimmune diseases and those requiring immunosuppression, such as patients undergoing organ transplantation, the strategy of expanding Tregs, such as IFN‐γ^+^FOXP3^+^ Tregs, ST2^+^FOXP3^+^ Tregs, and CD4^+^CXCR5^+^FOXP3^+^ Tfrs, may be highly effective. These strategies will hopefully be further improved and clinically validated to, be extended to the prevention of human immunological diseases.

**TABLE 1:** The primary functions of different subpopulations of FOXP3+ Tregs in human cancers, and their frequencies of leading to diseases exacerbation

Diseases	Markers	Primary Functions	The frequencies of Tregs of leading to disease exacerbation	References
Colorectal cancer	GZMB^+^ Tregs	GZMB^+^ Tregs could induce the apoptosis of effector T cells and were related to immune homeostasis and mediating tumor immunity.	↑	^[^ ^60^, ^61^, ^62^, ^63^ ^]^
	GZMB^+^TIM3^+^ Tregs	GZMB^+^TIM3^+^ Tregs presented higher cytolytic capacity towards autologous conventional T cells.	↑	^[^ ^65^, ^66^, ^67^ ^]^
	LAG3^+^TIM3^+^ Tregs	LAG3^+^TIM3^+^ Tregs presented significantly higher expression of TGF‐β, IL‐10, and CTLA‐4, which could suppress the expression of MHC‐II, CD80/CD86, and TNF‐α and increase the expression of IL‐10 in macrophages.	↑	^[^ ^12^, ^68^, ^69^ ^]^
	CCR8^+^ Tregs	The mAb therapy targeting CCR8^+^ Tregs could obviously inhibit tumor growth and improve the prognosis in CRCs by increasing tumor‐specific T cells.	↑	^[^ ^77^ ^]^
	BLIMP‐1^+^ eTregs	CRCs patients with low FOXP3^+^BLIMP‐1^+^ eTregs infiltration in tumors are associated with high risk of recurrence.	↓	^[^ ^80^ ^]^
	TNFR2^+^ Tregs	Blockade of TNFR2 signaling could markedly reduce the tumor‐infiltrating TNFR2^+^ Tregs in CRCs, whereas the number of tumor‐infiltrating IFN‐γ^+^CD8^+^ cytotoxic T lymphocytes were significantly increased.	↑	^[^ ^88^ ^]^
	CD30^+^OX40^+^ Tregs	High density of tumor‐infiltrating CD30^+^OX40^+^ Tregs in CRCs patients was associated with improved prognosis.	↓	^[^ ^93^ ^]^
Lung cancer	CD62L^high^CD25^+^CD4^+^ Tregs	CD62L^high^CD25^+^CD4^+^ Tregs could suppress cytokine production and inhibit the proliferation of Teffs.	↑	^[^ ^100^ ^]^
	GARP^+^ Tregs	GARP^+^ Tregs were highly infiltrated in tumor tissues in the early stage of lung cancer and exerted immunosuppressive effects through the GARP‐TGF‐β pathway to inhibit Teffs.	↑	^[^ ^112^ ^]^
	FOXA1^+^ Tregs	FOXA1^+^ Tregs could inhibit the antitumor immunity of T cells and promote tumor growth by the IFN‐β‐PI3K‐Akt‐FOXA1 signaling pathway.	↑	^[^ ^106^ ^]^
	FOXP3^+^ Helios^−^ Tregs	Higher percentages of tumor‐infiltrated FOXP3^+^ Helios^−^ Tregs were seen in advanced‐stage NSCLC with poorer survival.	↑	^[^ ^124^ ^]^
	TNFRSF9^+^ Tregs	TNFRSF9^+^ Tregs highly expressed immunosuppressive genes and were related to the poor prognosis of NSCLC.	↑	^[^ ^126^ ^]^
	TNFR2^+^ Tregs	The high percentage of TNFR2^+^ Tregs in the peripheral blood of lung cancer patients were associated with lymphatic invasion, distant metastasis, more advanced clinical stage, and worse outcomes.	↑	^[^ ^128^ ^]^
	CCR8^+^ Tregs	The high frequency of CCR8^+^ tumor‐infiltrating Tregs played a crucial suppressive function and was correlated with poor prognosis in patients with NSCLC.	↑	^[^ ^30^, ^129^ ^]^
	IRF4^+^ eTregs	The abundance of IRF4^+^ eTregs correlated with poor prognosis in patients with NSCLC.	↑	^[^ ^133^ ^]^
	CCR8^+^ICOS^+^ eTregs	The high expression of IRF4 was present in CCR8^+^ICOS^+^ eTregs, which further contributed to the superior suppressive activity and worse disease‐free survival and overall survival of NSCLC patients.	↑	^[^ ^133^ ^]^
	IL‐10^+^ and IL‐35^+^ Tregs	IL‐10^+^ and IL‐35^+^ Tregs‐derived IL‐10 and IL‐35 collectively induced inhibitory receptor, such as TIM‐3 and LAG‐3 expression on intratumoral CD8^+^ T cells, which further results in T‐cell dysfunction and antitumor immunity failure.	↑	^[^ ^138^ ^]^
Liver cancer	LAYN^+^FOXP3^+^Helios^+^ Tregs	The high expression of tumor‐infiltrated LAYN^+^FOXP3^+^Helios^+^ Tregs, which possessed suppressive functions, was associated with tumor‐infiltrating exhausted CD8^+^ T cells and poor survival in liver cancer.	↑	^[^ ^47^ ^]^
	LAYN^+^ Tregs	LAYN^+^ Tregs showed highly expressed LAYN, TNFRSF9, and ICOS, but the proportion of this cell population in tumor‐infiltrating cells was very small, which was different from FOXP3^+^ Tregs.	unknown	^[^ ^48^ ^]^
	GARP^+^/CTLA4^+^ Tregs	The high frequency of GARP^+^/CTLA4^+^ Tregs in patients with advanced HCC may facilitate immune dysregulation.	↑	
	CD4^+^CD69^+^FOXP3^−^ Tregs	CD4^+^CD69^+^FOXP3^−^ Tregs accounted for the vast majority of tumor‐infiltrating Tregs compared with FOXP3^+^ Tregs and could suppress the CD4^+^ T‐cell response mainly through mTGF‐β1.	↑	^[^ ^145^, ^146^]
	GITR^+^FOXP3^+^ Tregs	The expression of GITR was upregulated in activated tumor‐infiltrating Tregs in patients with primary or metastatic liver cancer, GITR ligation could abrogate the tumor‐infiltrating Tregs‐mediated suppression of effector T cells through treatment with soluble GITR ligand.	↑	^[^ ^147^ ^]^
	ICOS^+^ Tregs	ICOS^+^ Tregs could produce a mass of IL‐10 and TGF‐β1, higher ICOS^+^ Tregs levels and ICOS^+^ Tregs/CD4^+^ T cells ratios indicated worse prognosis in HCC.	↑	^[^ ^154^ ^]^
	CCR6^+^ Tregs	CCR6^+^ Tregs accounted for the majority of intratumoral Tregs, which could induced CD8^+^ T‐cell exhaustion and was associated with poor prognosis in patients with HCC	↑	^[^ ^159^ ^]^
	CCR4^+^ Tregs	The high frequency of CCR4^+^ Tregs exhibited potently immunosuppressive stem‐like specificity by upregulating TCF1, PD‐1, and CTLA‐4 levels and secreting more IL‐10 and IL‐35.	↑	^[^ ^161^ ^]^
	PD‐1^+^ eTregs	Enhanced PD‐1 expression on eTregs was observed in low‐glucose TME of HCC on account of PD‐1^+^ eTregs actively absorbed LA through MCT1 and promoted NFAT1 translocation into the nucleus.	↑	^[^ ^165^ ^]^
Gastric cancer	PD‐1^+^ eTregs	Tumor‐infiltrating eTregs highly expressed PD‐1 and that the proliferation and immunosuppressive activity of PD‐1^+^ eTregs could be reinforced by anti‐PD‐1 mAb therapy in patients with GC	↑	^[^ ^179^ ^]^
	VEGFR2^+^FOXP3^+^ eTregs	VEGFR2^+^FOXP3^+^ eTregs highly expressed Ki67 in GC tumor tissues	↑	^[^ ^192^ ^]^
	CD45RA^−^CCR7^−^ Tregs	Tumor‐infiltrating CD45RA−CCR7−Tregs were induced by tumor‐derived TNF‐α and could inhibit the secretion of IFN‐γ and proliferation of effector CD8^+^ T cells.	↑	^[^ ^198^ ^]^
	TNFR2^+^ Tregs	TNFR2^+^ Tregs preferentially accumulated in TME of GC, which expressed high levels of CTLA‐4 and CCR6 and possessed strong suppressive activity by activating TNF‐α/TNFR2 signaling pathway.	↑	^[^ ^199^ ^]^
Breast cancer	CCR8^+^ Tregs	Intratumoral CCR8^+^ Tregs highly expressed surface‐activated markers, such as CTLA‐4, CD39, and PD‐1, which contributed to the potential of immunosuppression.	↑	^[^ ^200^ ^]^
	OX40^+^ Tregs	Anti‐OX40 therapy could promote tumor‐infiltrated CD4^+^ T cells proliferation and reduce the tumor metastasis.	↑	^[^ ^209^ ^]^
Neck squamous cell carcinoma	Nrp1^+^ Tregs	Nrp1 expression in intratumoral Tregs appeared to correlate with poor prognosis in HNSCC	↑	^[^ ^206^ ^]^
	OX40^+^ Tregs	OX40 was particularly expressed on the surface of Tregs of HNSCC patients.	↑	^[^ ^207^ ^]^
	TIGIT^+^ Tregs	TIGIT^+^ Tregs could activate its ligand CD155, and the activation of TIGIT/CD155 signaling was associated with the pathologic grade and lymph node metastasis of HNSCC.	↑	^[^ ^231^ ^]^
Cutaneous squamous cell carcinoma	OX40^+^ Tregs	The application of anti‐OX40 could promote tumor‐infiltrated CD4^+^ T‐cell proliferation and reduce the tumor metastasis.	↑	^[^ ^208^ ^]^
Cervical cancer	Nrp1^+^ Tregs	The depletion of Nrp1^+^ Tregs in tumor‐draining lymph nodes was directly related to a favorable response to chemoradiotherapy in cervical cancer.	↑	^[^ ^205^ ^]^
	TNFR2^+^ Tregs	The proportion of TNFR2^+^ Tregs was found to be associated with the clinical stages of cervical cancer.	↑	^[^ ^211^ ^]^
Ovarian cancer	TNFR2^+^ Tregs	TNFR2^+^ Tregs mainly expressed high levels of CD39, CD73, TGF‐β, and GARP in the cell surface to increase their suppressive capacity, which further inhibited the production of IFN‐γ by Teffs.	↑	^[^ ^210^ ^]^
	IL‐17A^+^FOXP3^+^ Tregs	IL‐17A^+^FOXP3^+^ Tregs are a subpopulation of suppressive Tregs, which could be promoted by TGF‐β during tumor progression.	↑	^[^ ^216^ ^]^
Melanoma	Nrp1^+^ Tregs	Nrp1 expression in intratumoral Tregs appeared to correlate with poor prognosis in melanoma.	↑	^[^ ^206^ ^]^
	TIGIT^+^ Tregs	The high proportion of TIGIT^+^ Tregs and a high TIGIT/CD226 ratio in Tregs in the tumor immune microenvironment were associated with poor clinical prognosis in melanoma.	↑	^[^ ^228^ ^]^
Bladder cancer	TIGIT^+^ Tregs	TIGIT^+^ Tregs highly expressed IL‐32 and promoted the migration and invasion of tumor cells.	↑	^[^ ^229^ ^]^
Follicular lymphoma	TIGIT^+^ Tregs	The abundantly tumor‐infiltrating TIGIT^+^ Tregs showed enhanced suppressive capacity by inhibiting the activation and proliferation of CD8^+^ T cells in patients with follicular lymphoma.	↑	^[^ ^230^ ^]^

**TABLE 2:** The primary functions of different subpopulations of FOXP3+ Tregs in autoimmune diseases, transplantation, and pregnancy, and their frequencies of beneficial to diseases

Diseases	Markers	Primary functions	The frequencies of Tregs of beneficial to the disease	References
Systemic lupus erythematosus	CTLA‐4^+^ and GATA‐3^+^ Tregs	IL‐21 could suppress FOXP3^+^ Tregs differentiation and suppressive activity by inhibiting CTLA‐4 and GATA‐3 expression, whereas IL‐2 and TGF‐β promoted the expression of CTLA‐4^+^ Tregs and GATA‐3^+^ Tregs in SLE.	↑	^[^ ^236^ ^]^
	CD4^+^CXCR5^+^FOXP3^+^ Tfrs	The high proportion of circulating Tfrs could suppress Tfhs function and the frequency of Tfrs was positively associated with autoantibodies and might be a response to the enhanced humoral immunity during SLE pathogenesis.	↑	^[^ ^244^ ^]^
	PD‐1^+^ Tfrs	The high expression of PD‐1 was explored on Tfrs with dysfunction of suppressing Tfhs proliferation and activation in patients with SLE due to IL‐2 deficiency.	↓	^[^ ^246^ ^]^
	PSGL‐1^+^ Tfrs	PSGL‐1 interaction with selectin impaired the suppression function of Tfrs and contributed to the pathogenesis of SLE via inhibition of the TGF‐β pathway and reduced expression of FOXP3.	↓	^[^ ^250^ ^]^
Multiple sclerosis	CD39^+^FOXP3^+^ Tregs	CD39^+^FOXP3^+^ Tregs could suppress the production of IL‐17 by Th17 cells to alleviate the progression of MS.	↑	^[^ ^256^ ^]^
	FOXA1^+^ Tregs	FOXA1 could bind to PD‐L1, which was essential for FOXA1^+^ Tregs to kill activated T cells.	↑	^[^ ^118^ ^]^
	IFN‐γ^+^FOXP3^+^ Tregs	The suppressive activity of IFN‐γ^+^FOXP3^+^ Tregs was inhibited through the secretion of IFN‐γ stimulated by IL‐12 in MS.	↓	^[^ ^258^ ^]^
	CXCR5^+^PD‐1^+^FOXP3^+^CD25^+^ Tfrs	The lower frequency of circulating Tfrs was correlated to the reduction of IL‐10 that might increase the severity of MS.	↑	^[^ ^265^, ^266^, ^267^ ^]^
Rheumatoid arthritis	CD4^+^CXCR5^+^FOXP3^+^ Tfrs	The circulating CD4^+^CXCR5^+^FOXP3^+^ Tfrs with enhanced suppressive function could alleviate autoimmunity in RA patients by reducing IgG and IgM levels, and the proportion of Tfrs was negatively correlated with the disease severity.	↑	^[^ ^242^ ^]^
	TNFR2^+^ Tregs	TNF treatment could promote the proliferation of TNFR2^+^ Tregs, ameliorate inflammation by activation of TNF–TNFR2 signaling, and enhance expression of CD25.	↑	^[^ ^273^ ^]^
	TIM3^+^FOXP3^+^ Tregs	The proportion of TIM3^+^FOXP3^+^ Tregs was significantly decreased in patients with RA, which could potently suppress IFN‐γ and TNF‐α inflammation from Teffs by producing high IL‐10 and TGF‐β.	↑	^[^ ^276^ ^]^
Type 1 diabetes	CXCR5^+^FOXP3^+^ Tfrs	CXCR5^+^FOXP3^+^ Tfrs, which were needed to maintain peripheral tolerance by regulating diabetogenic Tfhs and B cells, were reduced in spleen and pancreatic lymph node of patients with T1D.	↑	^[^ ^279^ ^]^
	TIM1^+^ and TMI4^+^ Tregs	TIM1^+^ Tregs and TMI4^+^ Tregs were significantly decreased in PBMCs of patients with T1D. Inhibition of TIM1 could reduce FOXP3 expression and inhibit Tregs development, while TIM4 regulated the activation of naive T cells and proliferation of activated T cells.	↑	^[^ ^284^, ^285^, ^286^ ^]^
Organ transplantation	Notch‐1^+^ Tregs	Notch‐1^+^ Tregs were significantly higher in patients with a kidney transplant than in healthy controls, and inhibition of Notch‐1 could reduce the frequency and function of effector T cells, whereas the survival, proliferation, and suppressive function of Tregs were enhanced.	↓	^[^ ^291^ ^]^
	CXCR3^+^FOXP3^+^ Tregs	CXCR3^+^FOXP3^+^ Tregs showed the capacity to translocate to the site of inflammation and could control renal allograft rejection.	↑	^[^ ^293^ ^]^
Graft‐versus‐host disease	PD‐1^+^ Tregs	Low doses of IL‐2 enhanced PD‐1 expression of central memory Tregs in patients with cGVHD, suggesting that PD‐1 pathway was a critical homeostasis and tolerance regulator for Tregs.	↑	^[^ ^178^ ^]^
	TIM3^+^ Tregs	The subtype of TIM3^+^ Tregs exhibited highly immunosuppressive function in GVHD.	↑	^[^ ^303^ ^]^
	ST2^+^FOXP3^+^ Tregs	IL‐33 mediates the expansion of ST2^+^FOXP3^+^ Tregs via activation of p38 MAPK signaling after HSCT, which could protect against acute GVHD.	↑	^[^ ^307^ ^]^
	LAP^+^FOXP3^+^ Tregs	The subset of LAP^+^FOXP3^+^ Tregs possessed fully suppressive function and adoptive transfer of LAP^+^ Tregs could delay the development of xGVHD.	↑	^[^ ^308^ ^]^
	CD150^+^ Tregs	CD150^+^ Tregs could secret adenosine to maintain the quiescent status of HSCs, which activated the AMPK pathway to promote energy metabolism to inhibit the GVHD and intestinal cell apoptosis secondary to HSCT.	↑	^309^, ^310^
Pregnancy	Uterine Tregs	Uterine Tregs highly expressed IL1R2, LAYN, CD80, TNFRSF4, which were considered to be very important in establishing maternal‐fetal immune tolerance and indispensable for successful embryo implantation and pregnancy outcome.	↑	^317^
	PD‐1^high^IL‐10^+^ and TIGIT^+^FOXP3^low^Tregs	PD‐1^high^IL‐10^+^ and TIGIT^+^FOXP3^low^ Tregs could significantly inhibit CD4^+^ T‐cell proliferation.	↑	^318^
	CD25^high^HELIOS^+^FOXP3^+^ Tregs	CD25^high^HELIOS^+^FOXP3^+^ Tregs could simultaneously inhibit the proliferation of CD4^+^ and CD8^+^ Teffs and affect their production of certain cytokines.	↑	^318^

## AUTHOR CONTRIBUTIONS

Z.J., H.Z., P.W., and W.Q. wrote this manuscript and prepared the figure. F.D,. X.L., and L.Z. devised and supervised this project. All authors contributed to the article and approved the submitted version.

## CONFLICT OF INTEREST

The authors declare no conflict of interest.

## ETHICS APPROVAL

No ethical approval was required for this study.

## Data Availability

Not applicable.
